# [(Pyridine-2,6-dicarboxyl­ato)copper(II)]-μ-(pyridine-2,6-dicarboxyl­ato)-[bis­(ethyl­enediamine)­copper(II)]-μ-(pyridine-2,6-dicarboxyl­ato)-[(pyridine-2,6-dicarboxyl­ato)copper(II)] ethyl­enediamine monosolvate tetra­hydrate

**DOI:** 10.1107/S1600536812022039

**Published:** 2012-05-26

**Authors:** Amir Shokooh Saljooghi, Hadi Amiri Rudbari, Francesco Nicolò, Maliheh Zahmati, Fatemeh Delavar Mendi

**Affiliations:** a Department of Chemistry, Ferdowsi University of Mashhad, Mashhad 91779, Iran; b Dipartimento di Chimica Inorganica, Chimica Analitica e Chimica Fisica, Università di Messina, Salita Sperone, 31 Contrada Papardo, 98166 Messina, Italy

## Abstract

The title compound, [Cu_3_(C_7_H_3_NO_4_)_4_(C_2_H_8_N_2_)_2_]·C_2_H_8_N_2_·4H_2_O, was obtained by the reaction of copper(II) acetate dihydrate with pyridine-2,6-dicarb­oxy­lic acid (H_2_dipic) and ethyl­enediamine (en) in an aqueous solution. All of the Cu^II^ atoms in the trinuclear centrosymmetric title complex are six-coordinated in a distorted octa­hedral geometry with N_2_O_4_ and N_4_O_2_ environments for the outer and central Cu^II^ atoms, respectively. Various inter­actions, including numerous O—H⋯O and C—H⋯O hydrogen bonds and C—O⋯π stacking of the pyridine and carboxyl­ate groups [O⋯centroid distances = 3.669 (2) and 3.668 (2) Å] are observed in the crystal structure.

## Related literature

For metal complexes formed by pyridine­dicarb­oxy­lic acids, see: Aghabozorg *et al.* (2006 ▶); Burdock (1996 ▶); Douki *et al.* (2005 ▶); Kazuhiro *et al.* (1994 ▶); Murakami *et al.* (2003) ▶; Park *et al.* (2007 ▶); Xie *et al.* (2006 ▶). 
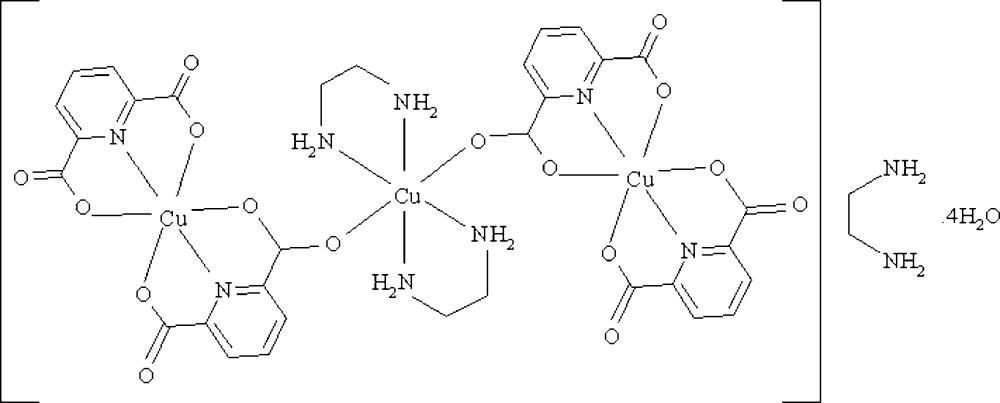


## Experimental

### 

#### Crystal data

[Cu_3_(C_7_H_3_NO_4_)_4_(C_2_H_8_N_2_)_2_]·C_2_H_8_N_2_·4H_2_O
*M* *_r_* = 1105.43
Monoclinic, 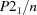

*a* = 8.152 (2) Å
*b* = 20.538 (5) Å
*c* = 12.736 (3) Å
β = 93.44 (2)°
*V* = 2128.5 (10) Å^3^
*Z* = 2
Mo *K*α radiation
μ = 1.58 mm^−1^
*T* = 293 K
0.51 × 0.28 × 0.12 mm

#### Data collection

Bruker APEXII CCD diffractometer
Absorption correction: multi-scan (*SADABS*; Bruker, 2007) ▶ *T*_min_ = 0.583, *T*_max_ = 0.747
35349 measured reflections
9453 independent reflections
6734 reflections with *I* > 2σ(*I*)
*R*_int_ = 0.034

#### Refinement

*R*[*F* ^2^ > 2σ(*F* ^2^)] = 0.035
*wR*(*F* ^2^) = 0.100
*S* = 1.02
9453 reflections
336 parameters
5 restraints
H atoms treated by a mixture of independent and constrained refinement
Δρ_max_ = 0.48 e Å^−3^
Δρ_min_ = −0.47 e Å^−3^

### 

Data collection: *APEX2* (Bruker, 2007 ▶); cell refinement: *SAINT* (Bruker, 2007 ▶); data reduction: *SAINT*; program(s) used to solve structure: *SHELXS97* (Sheldrick, 2008 ▶); program(s) used to refine structure: *SHELXL97* (Sheldrick, 2008 ▶); molecular graphics: *XP* in *SHELXTL* (Sheldrick, 2008 ▶); software used to prepare material for publication: *SHELXTL*.

## Supplementary Material

Crystal structure: contains datablock(s) I, global. DOI: 10.1107/S1600536812022039/qm2066sup1.cif

Structure factors: contains datablock(s) I. DOI: 10.1107/S1600536812022039/qm2066Isup2.hkl

Additional supplementary materials:  crystallographic information; 3D view; checkCIF report

## Figures and Tables

**Table 1:** Hydrogen-bond geometry (Å, °)

*D*—H⋯*A*	*D*—H	H⋯*A*	*D*⋯*A*	*D*—H⋯*A*
N4—H4*B*⋯O1	0.84 (2)	2.07 (2)	2.8697 (17)	160 (2)
O10—H10*B*⋯O2	0.92 (2)	1.87 (2)	2.7927 (17)	176 (3)
N5—H5*A*⋯O9	0.89	1.93	2.808 (2)	167
O9—H9*B*⋯O5	0.88 (2)	2.50 (2)	3.211 (2)	139 (3)
O9—H9*B*⋯O6	0.88 (2)	1.96 (2)	2.793 (2)	159 (3)
N3—H3*A*⋯O6^i^	0.87 (2)	2.51 (2)	3.218 (2)	138.5 (18)
O9—H9*A*⋯O10^ii^	0.83 (2)	2.10 (2)	2.893 (3)	160 (3)
N5—H5*B*⋯O9^iii^	0.89	2.59	3.106 (2)	118
N5—H5*B*⋯O10^iv^	0.89	2.06	2.9151 (19)	160
N5—H5*C*⋯O4^v^	0.89	1.82	2.6882 (18)	166
N4—H4*A*⋯O8^vi^	0.843 (19)	2.482 (19)	3.2079 (19)	144.8 (17)
O10—H10*A*⋯O8^vii^	0.88 (2)	1.82 (2)	2.6824 (17)	165 (2)
N3—H3*B*⋯O8^vii^	0.80 (2)	2.31 (3)	3.0664 (19)	156 (2)
N3—H3*B*⋯O7^vii^	0.80 (2)	2.43 (2)	3.1383 (17)	147 (2)

Symmetry codes: (i) 
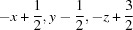
; (ii) 
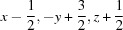
; (iii) 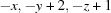; (iv) 
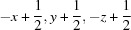
; (v) 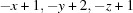; (vi) 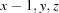; (vii) 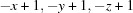.

**Fig. 1.**
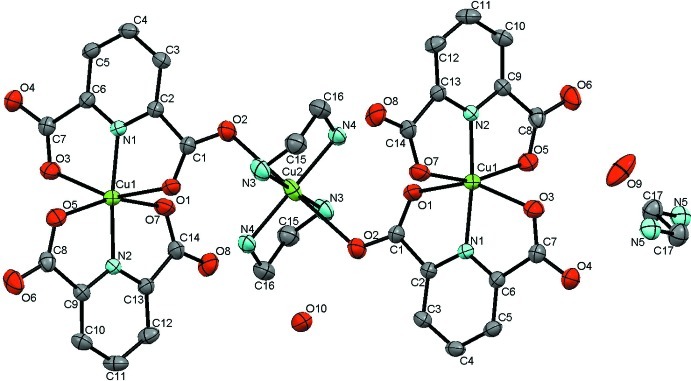
Molecular structure and atom labeling scheme for title compound with displacement ellipsoids at the 50% probability level.

**Fig. 2.**
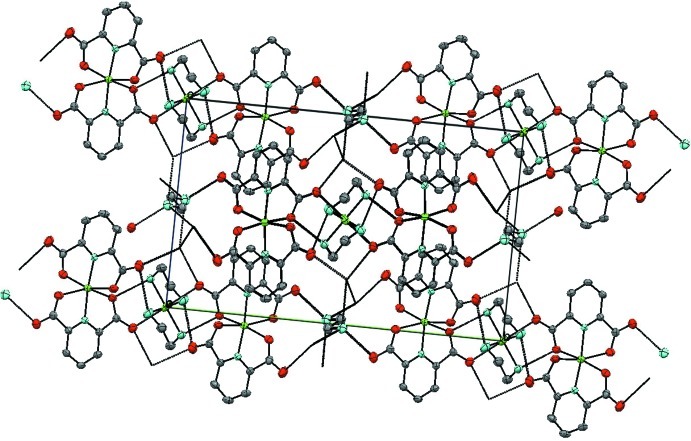
A packing view of the title compound. Hydrogen bonds are shown with dashed lines.

## supplementary crystallographic information

### Comment

Pyridine-2,6-dicarboxylic acid (also known as 2,6-dicarboxypyridine and as dipicolinic acid, H_2_dipic) is a water-soluble, commercially available, cheap and versatile N,*O*-chelator possessing diverse coordination modes, with a recognized biological function in the body metabolism (Douki *et al.*, 2005),and in a variety of processes as an enzyme inhibitor (Murakami *et al.*, 2003), plant preservative (Kazuhiro *et al.*, 1994), and food sanitizer (Burdock, 1996),. The complexation of transition metal ions from dipicolinic acid has been the subject of numerousreports. The reasons for this interest are the ability of the ligand so it gave stable chelates with different coordination modes (Park *et al.*, 2007), affinity to form strong hydrogen bonds and its biological activity in human metabolism (Xie *et al.*, 2006). A lots of dipicolinate complexes of transition metals and main groups are known and reported (Xie *et al.*, 2006; Aghabozorg *et al.*, 2006). Here, we report the crystal structure of the title binuclear complex, [Cu_3_(C_7_H_3_NO_4_)_4_(C_2_H_8_N_2_)_2_].(C_2_H_8_N_2_).2H_2_O, in which the Pyridine-2,6-dicarboxylic acid (C_7_H_3_NO_4_) species acts as a momo and tridentate ligand. In the crystal structure, the copper atoms are in three types. All copper atoms exhibit an distorted octahedral coordination geometry. In the crystal structure, Cu (1) is coordinated by two dipicolinic acid ligands, and Cu (2) is coordinated by two ethylenediamine molecules and two uncoordinated O atoms of two dipicolinic acid groups linked to Cu (1). As shown in Fig.1, this causes that Cu (2) makes a bridge between two Cu (1) atoms. The bond distances of Cu(1)– O(1) and Cu(1)– O(3) is longer than Cu(1)–N(1), Cu(1)–N(2), Cu(1)–O(5) and Cu(1)–O(7) bond distances due to Jahn–Teller effect. Also 0.74 Å difference between Cu(2)–O and Cu(2)–N bonds, due to Jahn–Teller effect. There are extensive intermolecular O—H···O, N—H···O and weak C—H···O hydrogen bonds, which cause the stability of the crystal structure (Fig. 2).

### Experimental

To an aqueous solution of Copper(II) acetate, an aqueous solution of H_2_dipic and en in 1:1:1 molar ratio was added. The final volume was 30 ml. After less than 1 h stirring, the obtained blue solution was left for 3 days. Then the blue crystals of the title compound were obtained for X-ray crystallography.

### Refinement

The H atoms of the water molecules were found in difference Fourier maps and the O–H bond lengths were constrained to 0.85 Å. The H atoms from C–H groups were placed in calculated positions. All H atoms were refined in riding model approximation with *U*_iso_(H) = 1.2*U*_eq_(C) or 1.5*U*_eq_(O).

### Crystal data

**Table:**

[Cu_3_(C_7_H_3_NO_4_)_4_(C_2_H_8_N_2_)_2_]·C_2_H_8_N_2_·4H_2_O	*F*(000) = 1134
*M**_r_* = 1105.43	*D*_x_ = 1.725 Mg m^−^^3^
Monoclinic, *P*2_1_/*n*	Mo *K*α radiation, λ = 0.71073 Å
Hall symbol: -P 2yn	Cell parameters from 8052 reflections
*a* = 8.152 (2) Å	θ = 2.6–32.5°
*b* = 20.538 (5) Å	µ = 1.58 mm^−^^1^
*c* = 12.736 (3) Å	*T* = 293 K
β = 93.44 (2)°	Irregular, blue
*V* = 2128.5 (10) Å^3^	0.51 × 0.28 × 0.12 mm
*Z* = 2	

### Data collection

**Table:**

Bruker APEXII CCD diffractometer	9453 independent reflections
Radiation source: fine-focus sealed tube	6734 reflections with *I* > 2σ(*I*)
Graphite monochromator	*R*_int_ = 0.034
φ and ω scans	θ_max_ = 35.2°, θ_min_ = 2.7°
Absorption correction: multi-scan (*SADABS*; Bruker, 2007)	*h* = −13→13
*T*_min_ = 0.583, *T*_max_ = 0.747	*k* = −33→33
35349 measured reflections	*l* = −20→20

### Refinement

**Table:**

Refinement on *F*^2^	Primary atom site location: structure-invariant direct methods
Least-squares matrix: full	Secondary atom site location: difference Fourier map
*R*[*F*^2^ > 2σ(*F*^2^)] = 0.035	Hydrogen site location: inferred from neighbouring sites
*wR*(*F*^2^) = 0.100	H atoms treated by a mixture of independent and constrained refinement
*S* = 1.02	*w* = 1/[σ^2^(*F*_o_^2^) + (0.0528*P*)^2^ + 0.1097*P*] where *P* = (*F*_o_^2^ + 2*F*_c_^2^)/3
9453 reflections	(Δ/σ)_max_ = 0.003
336 parameters	Δρ_max_ = 0.48 e Å^−^^3^
5 restraints	Δρ_min_ = −0.47 e Å^−^^3^

### Special details

**Table:**

Geometry. All s.u.'s (except the s.u. in the dihedral angle between two l.s. planes) are estimated using the full covariance matrix. The cell s.u.'s are taken into account individually in the estimation of s.u.'s in distances, angles and torsion angles; correlations between s.u.'s in cell parameters are only used when they are defined by crystal symmetry. An approximate (isotropic) treatment of cell s.u.'s is used for estimating s.u.'s involving l.s. planes.
Refinement. Refinement of *F*^2^ against ALL reflections. The weighted *R*-factor *wR* and goodness of fit *S* are based on *F*^2^, conventional *R*-factors *R* are based on *F*, with *F* set to zero for negative *F*^2^. The threshold expression of *F*^2^ > 2σ(*F*^2^) is used only for calculating *R*-factors(gt) *etc*. and is not relevant to the choice of reflections for refinement. *R*-factors based on *F*^2^ are statistically about twice as large as those based on *F*, and *R*- factors based on ALL data will be even larger.

### Fractional atomic coordinates and isotropic or equivalent isotropic displacement parameters (Å^2^)

**Table:**

	*x*	*y*	*z*	*U*_iso_*/*U*_eq_	
Cu1	0.41369 (2)	0.734887 (8)	0.553105 (12)	0.02551 (5)	
N1	0.38865 (14)	0.73453 (5)	0.39949 (8)	0.02225 (19)	
O5	0.26265 (15)	0.81147 (5)	0.60629 (9)	0.0362 (2)	
O3	0.58419 (15)	0.81797 (6)	0.50570 (8)	0.0368 (2)	
N2	0.44168 (14)	0.72476 (5)	0.70320 (8)	0.02212 (19)	
C2	0.29612 (16)	0.68844 (6)	0.35084 (10)	0.0236 (2)	
C6	0.46747 (16)	0.77861 (6)	0.34388 (10)	0.0233 (2)	
C9	0.36906 (16)	0.76619 (6)	0.76552 (10)	0.0229 (2)	
O1	0.21166 (16)	0.65984 (5)	0.51771 (8)	0.0374 (3)	
O2	0.15002 (16)	0.59160 (5)	0.38441 (9)	0.0401 (3)	
C13	0.53495 (16)	0.67598 (6)	0.74101 (10)	0.0240 (2)	
C10	0.38875 (18)	0.75991 (7)	0.87363 (11)	0.0302 (3)	
H10	0.3384	0.7889	0.9177	0.036*	
O7	0.58610 (16)	0.65866 (6)	0.56406 (8)	0.0401 (3)	
O6	0.21260 (16)	0.86312 (6)	0.75532 (11)	0.0468 (3)	
C3	0.27609 (18)	0.68619 (7)	0.24224 (10)	0.0270 (2)	
H3	0.2112	0.6542	0.2089	0.032*	
C1	0.21182 (18)	0.64213 (6)	0.42338 (11)	0.0277 (3)	
O4	0.62267 (16)	0.87514 (5)	0.36029 (9)	0.0413 (3)	
C5	0.45307 (18)	0.77855 (7)	0.23509 (10)	0.0276 (3)	
H5	0.5090	0.8091	0.1969	0.033*	
C7	0.56691 (17)	0.82794 (7)	0.40915 (11)	0.0269 (2)	
C4	0.35429 (19)	0.73235 (7)	0.18424 (11)	0.0296 (3)	
H4	0.3405	0.7323	0.1112	0.036*	
C11	0.48516 (19)	0.70947 (9)	0.91474 (11)	0.0353 (3)	
H11	0.5004	0.7044	0.9872	0.042*	
C12	0.55921 (18)	0.66641 (8)	0.84784 (11)	0.0312 (3)	
H12	0.6234	0.6321	0.8745	0.037*	
C8	0.27279 (17)	0.81864 (7)	0.70493 (12)	0.0290 (3)	
Cu2	0.0000	0.5000	0.5000	0.03467 (7)	
O8	0.70149 (17)	0.59043 (6)	0.68212 (10)	0.0447 (3)	
C14	0.61377 (19)	0.63770 (7)	0.65613 (12)	0.0297 (3)	
N4	0.06892 (19)	0.55367 (6)	0.62685 (10)	0.0333 (3)	
N3	0.2066 (2)	0.45052 (7)	0.53830 (13)	0.0394 (3)	
O10	0.2610 (2)	0.52256 (7)	0.21358 (10)	0.0516 (3)	
C15	0.3169 (2)	0.49316 (9)	0.60259 (14)	0.0394 (3)	
H15A	0.3641	0.5262	0.5591	0.047*	
H15B	0.4054	0.4681	0.6370	0.047*	
C16	0.2137 (3)	0.52425 (9)	0.68297 (13)	0.0442 (4)	
H16A	0.1795	0.4917	0.7324	0.053*	
H16B	0.2767	0.5574	0.7218	0.053*	
N5	0.28169 (16)	1.02440 (7)	0.51530 (11)	0.0352 (3)	
H5A	0.1986	1.0039	0.5433	0.053*	
H5B	0.2551	1.0331	0.4479	0.053*	
H5C	0.3024	1.0615	0.5498	0.053*	
O9	0.0509 (2)	0.94330 (9)	0.60524 (16)	0.0669 (5)	
C17	0.4283 (2)	0.98283 (8)	0.52329 (15)	0.0388 (3)	
H17A	0.4057	0.9423	0.4860	0.047*	
H17B	0.4562	0.9726	0.5966	0.047*	
H4A	−0.003 (2)	0.5641 (9)	0.6683 (15)	0.033 (5)*	
H3A	0.181 (3)	0.4176 (11)	0.5774 (18)	0.049 (6)*	
H4B	0.098 (3)	0.5904 (10)	0.6056 (18)	0.050 (6)*	
H3B	0.249 (3)	0.4324 (11)	0.491 (2)	0.054 (6)*	
H10A	0.273 (3)	0.4821 (7)	0.2364 (17)	0.055 (6)*	
H10B	0.228 (3)	0.5469 (10)	0.2693 (18)	0.079 (8)*	
H9A	−0.027 (3)	0.9446 (15)	0.645 (2)	0.082 (10)*	
H9B	0.095 (4)	0.9106 (12)	0.640 (2)	0.099 (11)*	

### Atomic displacement parameters (Å^2^)

**Table:**

	*U*^11^	*U*^22^	*U*^33^	*U*^12^	*U*^13^	*U*^23^
Cu1	0.03397 (10)	0.02360 (8)	0.01879 (7)	0.00059 (6)	0.00018 (6)	0.00041 (6)
N1	0.0263 (5)	0.0217 (4)	0.0186 (4)	−0.0015 (4)	0.0007 (4)	0.0018 (4)
O5	0.0444 (6)	0.0339 (5)	0.0293 (5)	0.0062 (5)	−0.0055 (4)	0.0029 (4)
O3	0.0466 (6)	0.0369 (5)	0.0262 (5)	−0.0096 (5)	−0.0049 (4)	0.0008 (4)
N2	0.0255 (5)	0.0208 (4)	0.0200 (4)	0.0013 (4)	0.0007 (4)	−0.0013 (4)
C2	0.0280 (6)	0.0219 (5)	0.0209 (5)	−0.0012 (4)	0.0006 (4)	−0.0001 (4)
C6	0.0249 (6)	0.0219 (5)	0.0232 (5)	−0.0001 (4)	0.0017 (4)	0.0024 (4)
C9	0.0227 (5)	0.0232 (5)	0.0228 (5)	0.0000 (4)	0.0015 (4)	−0.0028 (4)
O1	0.0582 (7)	0.0317 (5)	0.0227 (4)	−0.0143 (5)	0.0058 (5)	0.0006 (4)
O2	0.0550 (7)	0.0317 (5)	0.0341 (6)	−0.0186 (5)	0.0084 (5)	−0.0054 (4)
C13	0.0267 (6)	0.0227 (5)	0.0230 (5)	0.0017 (5)	0.0038 (5)	0.0012 (4)
C10	0.0312 (7)	0.0365 (7)	0.0233 (6)	0.0025 (6)	0.0040 (5)	−0.0056 (5)
O7	0.0601 (8)	0.0373 (6)	0.0235 (5)	0.0146 (5)	0.0066 (5)	−0.0020 (4)
O6	0.0555 (7)	0.0349 (6)	0.0494 (7)	0.0197 (5)	−0.0035 (6)	−0.0106 (5)
C3	0.0325 (7)	0.0269 (6)	0.0214 (5)	−0.0002 (5)	−0.0006 (5)	−0.0038 (5)
C1	0.0349 (7)	0.0232 (5)	0.0251 (6)	−0.0056 (5)	0.0029 (5)	0.0002 (5)
O4	0.0551 (7)	0.0306 (5)	0.0385 (6)	−0.0163 (5)	0.0059 (5)	0.0026 (5)
C5	0.0321 (7)	0.0289 (6)	0.0221 (5)	0.0011 (5)	0.0042 (5)	0.0061 (5)
C7	0.0276 (6)	0.0241 (5)	0.0290 (6)	−0.0013 (5)	0.0018 (5)	0.0007 (5)
C4	0.0358 (7)	0.0349 (7)	0.0181 (5)	0.0043 (6)	0.0024 (5)	0.0000 (5)
C11	0.0363 (8)	0.0512 (9)	0.0185 (5)	0.0044 (7)	0.0014 (5)	0.0025 (6)
C12	0.0319 (7)	0.0354 (7)	0.0264 (6)	0.0074 (6)	0.0021 (5)	0.0077 (5)
C8	0.0278 (6)	0.0244 (6)	0.0342 (7)	0.0022 (5)	−0.0038 (5)	−0.0017 (5)
Cu2	0.03599 (14)	0.03264 (13)	0.03617 (14)	0.00045 (10)	0.00872 (11)	−0.01548 (11)
O8	0.0584 (8)	0.0315 (5)	0.0455 (7)	0.0218 (5)	0.0139 (6)	0.0061 (5)
C14	0.0377 (7)	0.0231 (5)	0.0287 (6)	0.0051 (5)	0.0064 (5)	−0.0012 (5)
N4	0.0461 (7)	0.0260 (5)	0.0292 (6)	−0.0023 (5)	0.0151 (5)	−0.0052 (5)
N3	0.0467 (8)	0.0333 (7)	0.0395 (7)	0.0063 (6)	0.0122 (6)	−0.0102 (6)
O10	0.0888 (11)	0.0350 (6)	0.0313 (6)	0.0095 (7)	0.0053 (6)	0.0035 (5)
C15	0.0423 (9)	0.0407 (8)	0.0352 (8)	0.0023 (7)	0.0036 (7)	−0.0012 (7)
C16	0.0643 (12)	0.0405 (8)	0.0279 (7)	0.0044 (8)	0.0033 (7)	−0.0041 (6)
N5	0.0316 (6)	0.0360 (6)	0.0377 (7)	−0.0032 (5)	−0.0007 (5)	−0.0016 (5)
O9	0.0400 (7)	0.0670 (10)	0.0921 (12)	−0.0022 (7)	−0.0090 (8)	0.0472 (9)
C17	0.0366 (8)	0.0355 (7)	0.0447 (9)	−0.0001 (6)	0.0070 (7)	0.0026 (7)

### Geometric parameters (Å, º)

**Table:**

Cu1—N2	1.9228 (11)	C4—H4	0.9300
Cu1—N1	1.9549 (11)	C11—C12	1.391 (2)
Cu1—O7	2.1030 (11)	C11—H11	0.9300
Cu1—O5	2.1328 (11)	C12—H12	0.9300
Cu1—O1	2.2808 (11)	Cu2—N3^i^	2.0019 (16)
Cu1—O3	2.3041 (11)	Cu2—N3	2.0019 (16)
N1—C6	1.3375 (16)	Cu2—N4	2.0080 (13)
N1—C2	1.3390 (17)	Cu2—N4^i^	2.0080 (13)
O5—C8	1.2626 (18)	O8—C14	1.2389 (17)
O3—C7	1.2461 (18)	N4—C16	1.473 (2)
N2—C9	1.3271 (16)	N4—H4A	0.843 (19)
N2—C13	1.3303 (16)	N4—H4B	0.84 (2)
C2—C3	1.3837 (18)	N3—C15	1.469 (2)
C2—C1	1.5188 (18)	N3—H3A	0.87 (2)
C6—C5	1.3834 (18)	N3—H3B	0.80 (2)
C6—C7	1.5146 (19)	O10—H10A	0.883 (15)
C9—C10	1.3826 (19)	O10—H10B	0.921 (16)
C9—C8	1.5162 (19)	C15—C16	1.505 (2)
O1—C1	1.2553 (17)	C15—H15A	0.9700
O2—C1	1.2439 (17)	C15—H15B	0.9700
C13—C12	1.3772 (19)	C16—H16A	0.9700
C13—C14	1.5109 (18)	C16—H16B	0.9700
C10—C11	1.384 (2)	N5—C17	1.467 (2)
C10—H10	0.9300	N5—H5A	0.8900
O7—C14	1.2567 (18)	N5—H5B	0.8900
O6—C8	1.2350 (18)	N5—H5C	0.8900
C3—C4	1.381 (2)	O9—H9A	0.831 (17)
C3—H3	0.9300	O9—H9B	0.875 (17)
O4—C7	1.2520 (17)	C17—C17^ii^	1.516 (3)
C5—C4	1.380 (2)	C17—H17A	0.9700
C5—H5	0.9300	C17—H17B	0.9700
			
N2—Cu1—N1	173.52 (4)	C10—C11—H11	119.9
N2—Cu1—O7	79.31 (4)	C12—C11—H11	119.9
N1—Cu1—O7	95.33 (4)	C13—C12—C11	118.20 (13)
N2—Cu1—O5	78.49 (4)	C13—C12—H12	120.9
N1—Cu1—O5	107.04 (4)	C11—C12—H12	120.9
O7—Cu1—O5	157.55 (4)	O6—C8—O5	126.80 (14)
N2—Cu1—O1	99.47 (4)	O6—C8—C9	118.08 (13)
N1—Cu1—O1	76.64 (4)	O5—C8—C9	115.12 (12)
O7—Cu1—O1	88.96 (5)	N3^i^—Cu2—N3	180.0
O5—Cu1—O1	97.89 (5)	N3^i^—Cu2—N4	96.20 (6)
N2—Cu1—O3	107.65 (4)	N3—Cu2—N4	83.80 (6)
N1—Cu1—O3	76.64 (4)	N3^i^—Cu2—N4^i^	83.80 (6)
O7—Cu1—O3	99.06 (5)	N3—Cu2—N4^i^	96.20 (6)
O5—Cu1—O3	84.60 (5)	N4—Cu2—N4^i^	180.00 (6)
O1—Cu1—O3	152.68 (4)	O8—C14—O7	125.87 (13)
C6—N1—C2	120.58 (11)	O8—C14—C13	118.69 (13)
C6—N1—Cu1	120.18 (9)	O7—C14—C13	115.42 (12)
C2—N1—Cu1	119.20 (8)	C16—N4—Cu2	110.04 (10)
C8—O5—Cu1	113.42 (9)	C16—N4—H4A	111.8 (13)
C7—O3—Cu1	110.25 (9)	Cu2—N4—H4A	118.2 (13)
C9—N2—C13	122.16 (11)	C16—N4—H4B	106.9 (15)
C9—N2—Cu1	119.64 (9)	Cu2—N4—H4B	107.7 (16)
C13—N2—Cu1	118.20 (8)	H4A—N4—H4B	101.2 (19)
N1—C2—C3	121.04 (12)	C15—N3—Cu2	108.13 (10)
N1—C2—C1	115.09 (11)	C15—N3—H3A	107.5 (15)
C3—C2—C1	123.82 (12)	Cu2—N3—H3A	108.0 (14)
N1—C6—C5	121.00 (12)	C15—N3—H3B	114.6 (17)
N1—C6—C7	114.86 (11)	Cu2—N3—H3B	116.4 (17)
C5—C6—C7	124.12 (12)	H3A—N3—H3B	101 (2)
N2—C9—C10	120.42 (12)	H10A—O10—H10B	106.7 (17)
N2—C9—C8	112.77 (11)	N3—C15—C16	106.47 (15)
C10—C9—C8	126.78 (12)	N3—C15—H15A	110.4
C1—O1—Cu1	110.04 (9)	C16—C15—H15A	110.4
N2—C13—C12	120.68 (12)	N3—C15—H15B	110.4
N2—C13—C14	113.03 (11)	C16—C15—H15B	110.4
C12—C13—C14	126.20 (12)	H15A—C15—H15B	108.6
C9—C10—C11	118.42 (12)	N4—C16—C15	107.96 (13)
C9—C10—H10	120.8	N4—C16—H16A	110.1
C11—C10—H10	120.8	C15—C16—H16A	110.1
C14—O7—Cu1	113.50 (9)	N4—C16—H16B	110.1
C4—C3—C2	118.76 (13)	C15—C16—H16B	110.1
C4—C3—H3	120.6	H16A—C16—H16B	108.4
C2—C3—H3	120.6	C17—N5—H5A	109.5
O2—C1—O1	126.87 (13)	C17—N5—H5B	109.5
O2—C1—C2	117.84 (12)	H5A—N5—H5B	109.5
O1—C1—C2	115.30 (11)	C17—N5—H5C	109.5
C4—C5—C6	118.86 (12)	H5A—N5—H5C	109.5
C4—C5—H5	120.6	H5B—N5—H5C	109.5
C6—C5—H5	120.6	H9A—O9—H9B	91 (3)
O3—C7—O4	126.50 (14)	N5—C17—C17^ii^	110.25 (17)
O3—C7—C6	117.18 (12)	N5—C17—H17A	109.6
O4—C7—C6	116.32 (13)	C17^ii^—C17—H17A	109.6
C5—C4—C3	119.72 (12)	N5—C17—H17B	109.6
C5—C4—H4	120.1	C17^ii^—C17—H17B	109.6
C3—C4—H4	120.1	H17A—C17—H17B	108.1
C10—C11—C12	120.11 (13)		
			
O7—Cu1—N1—C6	−100.17 (11)	C8—C9—C10—C11	−178.36 (14)
O5—Cu1—N1—C6	77.88 (11)	N2—Cu1—O7—C14	6.88 (11)
O1—Cu1—N1—C6	172.17 (11)	N1—Cu1—O7—C14	−169.43 (11)
O3—Cu1—N1—C6	−2.10 (10)	O5—Cu1—O7—C14	15.4 (2)
O7—Cu1—N1—C2	77.69 (10)	O1—Cu1—O7—C14	−92.96 (12)
O5—Cu1—N1—C2	−104.25 (10)	O3—Cu1—O7—C14	113.29 (12)
O1—Cu1—N1—C2	−9.96 (10)	N1—C2—C3—C4	0.3 (2)
O3—Cu1—N1—C2	175.77 (11)	C1—C2—C3—C4	177.64 (13)
N2—Cu1—O5—C8	6.16 (10)	Cu1—O1—C1—O2	160.26 (14)
N1—Cu1—O5—C8	−177.35 (10)	Cu1—O1—C1—C2	−20.20 (16)
O7—Cu1—O5—C8	−2.43 (19)	N1—C2—C1—O2	−166.87 (13)
O1—Cu1—O5—C8	104.28 (11)	C3—C2—C1—O2	15.7 (2)
O3—Cu1—O5—C8	−103.11 (11)	N1—C2—C1—O1	13.55 (19)
N2—Cu1—O3—C7	−177.91 (10)	C3—C2—C1—O1	−163.89 (14)
N1—Cu1—O3—C7	7.14 (10)	N1—C6—C5—C4	0.6 (2)
O7—Cu1—O3—C7	100.50 (10)	C7—C6—C5—C4	−177.81 (13)
O5—Cu1—O3—C7	−101.83 (10)	Cu1—O3—C7—O4	169.07 (13)
O1—Cu1—O3—C7	−5.07 (16)	Cu1—O3—C7—C6	−10.36 (15)
O7—Cu1—N2—C9	174.33 (11)	N1—C6—C7—O3	9.37 (19)
O5—Cu1—N2—C9	−2.34 (10)	C5—C6—C7—O3	−172.16 (14)
O1—Cu1—N2—C9	−98.55 (10)	N1—C6—C7—O4	−170.12 (13)
O3—Cu1—N2—C9	78.13 (11)	C5—C6—C7—O4	8.4 (2)
O7—Cu1—N2—C13	−5.37 (10)	C6—C5—C4—C3	−1.9 (2)
O5—Cu1—N2—C13	177.95 (11)	C2—C3—C4—C5	1.5 (2)
O1—Cu1—N2—C13	81.74 (10)	C9—C10—C11—C12	−0.2 (2)
O3—Cu1—N2—C13	−101.58 (10)	N2—C13—C12—C11	−0.7 (2)
C6—N1—C2—C3	−1.7 (2)	C14—C13—C12—C11	175.45 (14)
Cu1—N1—C2—C3	−179.57 (10)	C10—C11—C12—C13	0.6 (2)
C6—N1—C2—C1	−179.23 (12)	Cu1—O5—C8—O6	171.92 (14)
Cu1—N1—C2—C1	2.91 (15)	Cu1—O5—C8—C9	−8.36 (16)
C2—N1—C6—C5	1.2 (2)	N2—C9—C8—O6	−173.61 (13)
Cu1—N1—C6—C5	179.08 (10)	C10—C9—C8—O6	4.7 (2)
C2—N1—C6—C7	179.77 (11)	N2—C9—C8—O5	6.65 (18)
Cu1—N1—C6—C7	−2.39 (15)	C10—C9—C8—O5	−175.07 (14)
C13—N2—C9—C10	0.1 (2)	Cu1—O7—C14—O8	174.91 (13)
Cu1—N2—C9—C10	−179.56 (10)	Cu1—O7—C14—C13	−6.97 (17)
C13—N2—C9—C8	178.54 (11)	N2—C13—C14—O8	−178.92 (13)
Cu1—N2—C9—C8	−1.15 (15)	C12—C13—C14—O8	4.7 (2)
N2—Cu1—O1—C1	−157.62 (10)	N2—C13—C14—O7	2.82 (19)
N1—Cu1—O1—C1	17.09 (10)	C12—C13—C14—O7	−173.59 (15)
O7—Cu1—O1—C1	−78.64 (11)	N3^i^—Cu2—N4—C16	173.36 (12)
O5—Cu1—O1—C1	122.82 (10)	N3—Cu2—N4—C16	−6.64 (12)
O3—Cu1—O1—C1	29.29 (16)	N4—Cu2—N3—C15	−22.24 (11)
C9—N2—C13—C12	0.3 (2)	N4^i^—Cu2—N3—C15	157.76 (11)
Cu1—N2—C13—C12	−179.98 (11)	Cu2—N3—C15—C16	45.93 (16)
C9—N2—C13—C14	−176.31 (12)	Cu2—N4—C16—C15	33.54 (17)
Cu1—N2—C13—C14	3.39 (15)	N3—C15—C16—N4	−52.17 (18)
N2—C9—C10—C11	−0.2 (2)		

Symmetry codes: (i) −*x*, −*y*+1, −*z*+1; (ii) −*x*+1, −*y*+2, −*z*+1.

### Hydrogen-bond geometry (Å, º)

**Table:**

*D*—H···*A*	*D*—H	H···*A*	*D*···*A*	*D*—H···*A*
N4—H4*B*···O1	0.84 (2)	2.07 (2)	2.8697 (17)	160 (2)
O10—H10*B*···O2	0.92 (2)	1.87 (2)	2.7927 (17)	176 (3)
N5—H5*A*···O9	0.89	1.93	2.808 (2)	167
O9—H9*B*···O5	0.88 (2)	2.50 (2)	3.211 (2)	139 (3)
O9—H9*B*···O6	0.88 (2)	1.96 (2)	2.793 (2)	159 (3)
N3—H3*A*···O6^iii^	0.87 (2)	2.51 (2)	3.218 (2)	138.5 (18)
O9—H9*A*···O10^iv^	0.83 (2)	2.10 (2)	2.893 (3)	160 (3)
N5—H5*B*···O9^v^	0.89	2.59	3.106 (2)	118
N5—H5*B*···O10^vi^	0.89	2.06	2.9151 (19)	160
N5—H5*C*···O4^ii^	0.89	1.82	2.6882 (18)	166
N4—H4*A*···O8^vii^	0.843 (19)	2.482 (19)	3.2079 (19)	144.8 (17)
O10—H10*A*···O8^viii^	0.88 (2)	1.82 (2)	2.6824 (17)	165 (2)
N3—H3*B*···O8^viii^	0.80 (2)	2.31 (3)	3.0664 (19)	156 (2)
N3—H3*B*···O7^viii^	0.80 (2)	2.43 (2)	3.1383 (17)	147 (2)

Symmetry codes: (ii) −*x*+1, −*y*+2, −*z*+1; (iii) −*x*+1/2, *y*−1/2, −*z*+3/2; (iv) *x*−1/2, −*y*+3/2, *z*+1/2; (v) −*x*, −*y*+2, −*z*+1; (vi) −*x*+1/2, *y*+1/2, −*z*+1/2; (vii) *x*−1, *y*, *z*; (viii) −*x*+1, −*y*+1, −*z*+1.
